# Genetic Diversity and Structure of Domestic Cavy (*Cavia porcellus*) Populations from Smallholder Farms in Southern Cameroon

**DOI:** 10.20884/1.jap.2017.19.1.585

**Published:** 2017

**Authors:** Basengere Ayagirwe, Felix Meutchieye, Appolinaire Djikeng, Robert Skilton, Sarah Osama, Yacouba Manjeli

**Affiliations:** 1 Department of Animal Production, Faculty of Agriculture and Environmental Studies, Evangelical University in Africa, Bukavu, The Democratic Republic of the Congo; 2 Department of Animal Production, Faculty of Agronomy and Agricultural Sciences, University of Dschang, Cameroon; 3 Biosciences Eastern and Central Africa - International Livestock Research Institute (BecA-ILRI), Nairobi, Kenya; 4 International Centre of Insect Physiology and Ecology (icipe), Nairobi, Kenya

**Keywords:** guinea pig, microsatellites, genetic diversity, small stock, sub-Saharan Africa, marmut, mikro satelit, keragaman genetik, skala kecil, sub-Sahara Afrika

## Abstract

Although domestic cavies are widely used in sub-Saharan Africa as a source of meat and income, there are only a few studies of their population structure and genetic relatedness. This seminal study was designed with the main objective to assess the genetic diversity and determine the population structure of cavy populations from Cameroon to guide the development of a cavy improvement program. Sixteen microsatellite markers were used to genotype 109 individuals from five cavy populations (Wouri, Moungo and Nkongsamba in the Littoral region, and Mémé and Fako in the Southwest region of Cameroon). Twelve markers worked in the five populations with a total of 17 alleles identified, with a range of 2.9 to 4.0 alleles per locus. Observed heterozygosity (from 0.022 to 0.277) among populations was lower than expected heterozygosity (from 0.42 to 0.54). Inbreeding rates between individuals of the populations and between individuals in each population were 59.3% and 57.2%, respectively, against a moderate differentiation rate of 4.9%. All the tested loci deviated from Hardy-Weinberg equilibrium, except for locus 3. Genetic distances between populations were small (from 0.008 to 0.277), with a high rate of variability among individuals within each population (54.4%). Three distinct genetic groups were structured. This study has shown that microsatellites are useful for the genetic characterization of cavy populations in Cameroon and that the populations investigated have sufficient genetic diversity that can be used to be deployed as a basis for weight, prolificacy and disease resistance improvement. The genetic of diversity in Southern Cameroon is wide and constitute an opportunity for cavy breeding program.

## Abstract (id)

Marmut banyak diternakkan di daerah sub-Sahara Afrika sebagai sumber daging dan pendapatan, namun studi tentang struktur populasi dan hubungan genetik pada marmut masih terbatas. Tujuan penelitian ini adalah untuk mengetahui keragaman genetik dan struktur populasi marmut di Kamerun sebagai panduan dalam pengembangan populasi marmut. Sebanyak 16 penanda mikrosatelit digunakan untuk mengidentifikasi genotip dari 109 individu di lima daerah (Wouri, Moungo dan Nkongsamba di Littoral region, dan Mémé serta Fako di daerah Baratdaya Kamerun). Sebanyak 12 marker dapat digunakan untuk mengidentifikasi 17 allel di lima daerah populasi dengan kisaran 2,9 sampai 4,0 allel per lokus. Heterozigositas yang diamati dalam populasi (0,022 sampai 0,277) ternyata lebih rendah daripada yang diharapkan (0,42 sampai 0,54). Laju inbreeding antar individu dalam populasi dan antar individu di masing-masing populasi berturut-turut adalah 59,3% dan 57,2%, dengan laju perbedaan yang sedang yakni 4,9%. Semua loci yang diuji memiliki deviasi dari keseimbangan Hardy-Weinberg, kecuali pada lokus 3. Jarak genetik antar populasi menunjukkan jarak yang kecil (0,008 sampai 0,277), dengan variasi yang tinggi antar individu di tiap populasi (54,4%). Tiga kelompok genetik dapat diketahui. Studi ini menunjukkan bahwa mikro satelit bermanfaat untuk melakukan karakterisasi genetik pada populasi marmut di Kamerun dan populasi yang dipelajari memiliki keragaman genetik yang memadai sehingga dapat digunakan sebagai basis untuk bobot, prolifikasi dan peningkatan ketahanan penyakit. Keragaman genetik di Baratdaya Kamerun sangat tinggi dan membuka peluang untuk program breeding marmut.

## Introduction

Cavy culture has demonstrated its utility and effectiveness for poverty alleviation in Andean countries in South America (Chauca 1997). However, in numerous sub-Saharan African countries considerable populations of domestic cavies (*Cavia porcellus* L.) exist that serve for meat consumption, income generation and manure production in mixed crop-livestock systems (Ngou Ngoupayou et al. 1995; Kouakou et al. 2011; Matthiesen et al. 2011; Niba et al. 2012; Maass et al. 2014; Yiva et al. 2014).

It is not known, where, when and how often cavies have been introduced in Cameroon. However, it is speculated that cavy husbandry has been known at least since 1968 (Yiva et al. 2014). Except for two development projects (Hardouin 1995; Nuwanyakpa et al. 1997), there was no promotion of the animal, but farmers took up cavy culture by themselves across the country. To increase productivity, one project introduced descendants from a large body size line developed in Peru to Northwest Cameroon in 1996 (Nuwanyakpa et al. 1997). Niba et al. (2012), however, presume that this breed has possibly been assimilated in the local gene pool.

Since 2001, the use of cavies has been promoted by the Ministry of Livestock, Fisheries and Animal Industries in Cameroon as part of short-cycle livestock species targeted to meet the national demand for animal proteins (Niba et al. 2012). Domestic cavies are widely reared and consumed in Cameroon (Manjeli et al. 1998; Ngou Ngoupayou et al. 1995; Niba et al. 2012; Yiva et al. 2014), contributing to income generation in poor households since their rearing does not require significant capital (Lammers et al. 2009; Niba et al. 2012).

Although cavies appear morphologically similar considering their coat color pattern, genetically they might represent different populations. Largest cavy populations are known to exist in Cameroon with high phenotypic viabilities (Ayagirwe et al. 2015); this have been studied by molecular markers (Poutougnigni et al. 2015; Wikondi et al. 2015) in some populations and did not cover the southern part of the country. Ayagirwe (2014) suggested that the main ecotypes in southern Cameroon are from Wouri, Moungo and Nkongsamba in the Littoral (coastal) region, and Fako and Mémé in the Southwest region.

Because of their polymorphism and their homogeneous distribution in the genome, molecular markers are powerful tools for describing animal populations (Mohamed et al. 2010). However, the use of molecular markers in domestic cavy to reveal diversity patterns is recent and limited to few populations from South America (Spotorno et al. 2004; Kanitz et al. 2009; Burgos et al. 2011) and, only lately, from Africa for the Côte d’Ivoire cavy population (Kouakou et al. 2015). It is, therefore, necessary to extend these studies to populations in other African countries for better understanding cavy genetic diversity to help to guide cavy breeding in sub-Saharan Africa.

## Materials and Methods

### Study area

This study was conducted in Wouri, Moungo and Nkongsamba in the Littoral (coastal) region, and Mémé and Fako in the Southwest region of Cameroon. The Littoral and Southwest administrative regions of Cameroon, a unimodal agro-ecological zone, are located between 2°6' and 6°12' north, 8°48' and 10°30' east, with elevations from 0 to 4095 m a.s.l. This region occupies an area of 4.5 million ha, of which 282,000 ha (6.3%) are cultivated with agricultural crops. The climate is typically equatorial: with an average annual rainfall of 2500 to 4000 mm, air humidity of 85 to 90%, and mean monthly temperature between 22 and 29°C (FAO 2008).

### Sampling and collection of blood

A total of 109 blood samples were collected from adult cavies of about four months of age.To select within the five populations, the snowball method was used (Salganik and Heckathorn 2004), because of lack of information available on cavy keepers. Identified cavy-keeping household helped in identifying others. Usually, one animal was sampled if the cavy population size per household was below 30 animals, except if conspicuously unusual phenotypes were encountered. The animals were selected with the objective of obtaining the largest variability of phenotypic characteristics within each population following the method of Kosaki and Juo (1989).

Blood samples were collected from adult animals by pricking the disinfected ear vein with a 22 gauge needle. Blood was spotted onto Whatman FTA Classic Cards (Cambridge Bioscience, United Kingdom), according to the manufacturer’s instructions and air dried.

### DNA extraction

For the total DNA extraction, twenty 1.5 mm punches of the FTA Card blood spots were added to 1 ml of 100 mM Tris pH 8, 0.1% SDS and the supernatant was removed after 30 min of agitation. A total volume of five hundred µl of 1.5 M guanidinium thiocyanate was added, and after 10 min of vortexing all the supernatant was removed. The discs were then washed three times with 500 µl of triple distilled water, with 10 min of centrifugation between each wash. Then, 50 µl triple distilled water was added to the discs and the mixture placed in a water bath and heated to 90 °C for 20 min. After 30 min of cooling, DNA was recovered by centrifugation and stored at 20°C.

### PCR amplification of microsatellites

Sixteen microsatellite markers previously reported for the species *Cavia porcellus* were evaluated (Spotorno et al. 2004; Kanitz et al. 2009; Burgos et al. 2011). PCR amplification was carried out in C1000 thermal cycler (Bio-Rad). The final volume of the reaction was 10 µl, containing 2 µl of 40 ng/µl template DNA, 1x DreamTaq buffer containing 2 mM MgCl_2_ (Fermentas), 200 μM of each dNTP, and 0.4 U of DreamTaq DNA polymerase (Fermentas). PCR conditions were: 95°C for 3 min, followed by 35 cycles of 30 sec at 94°C for template denaturation, 30 sec at 60°C primer annealing and 60 sec at 72°C for primer extension, followed a final primer extension step at 72 °C for 20 min. PCR products were analyzed using 1.5% agarose gel electrophoresis.

### Data analysis

The data was captured using the GenScan ® collection software (Applied Biosystems) and the allelic data analyzed using the GeneMapper ® software version 4.1 (Applied biosystems). A total 103 data points were achieved out of the expected 109 data point giving an overall success rate of 94.5 % .The data was compiled into a spreadsheet as a standard GeneMapper output file and used in subsequent analysis.

Allele scoring was done using GeneMapper software V4.1 (Timothy 2009). The observed heterozygosity (Ho) and unbiased expected heterozygosity (He) were calculated under the assumption of Hardy-Weinberg equilibrium with allele frequencies observed according to Nei (1978); the average number of alleles (Na) (Crow and Kimura 1970) were calculated using Arlequin software V3.1 (Excoffier et al. 2005). The test of Hardy-Weinberg equilibrium was conducted with Power Marker software V3.25 (Liu and Muse 2005).

Admixture were investigated. For grouping individuals into a K^th^ number of population, Bayesian probabilistic group assignment was done using STRUCTURE software (Pritchard et al. 2000). K values analyzed ranged from to 1-5 and each one was simulated five times. Correlated allele frequency with mixing model was used for runs with 100,000 iteration following a 10,000 burn-in period. The Delta K method described by Evanno et al. (2005) was applied for inferring optimal k-values.

## Results and Discussion

### Indices of genetic diversity within and among populations

Mean number of alleles observed varied from 2.909 ± 1.221 for the population of Nkongsamba to 4.0 ± 1.549 for the population of Wouri, with an overall average of 3.455 ± 1.809 (Table 1). The lower number of alleles found for the population of Nkongsamba may partly reflect geographical differences observed between different areas, but also the smaller sample size compared to the other populations.

**Table 1:** Number of alleles and heterozygosity rate in five cavy populations studied from Littoral and Southwest Regions of Cameroon (N = 109)

Locus	Wouri (N = 21)			Nkongsamba (N = 15)			Moungo (N = 30)			Mémé (N = 22)			Fako (N = 21)			
	Na	Ho	He	Na	Ho	He	Na	Ho	He	Na	Ho	He	Na	Ho	He	F
*Cavy*2	na	na	na	3	0.083	0.163	2	0.037	0.107	2	0.000	0.138	2	0.111	0.203	0.72
*Cavy*3	2	0.125	0.121	2	0.071	0.071	na	na	na	na	na	na	na	na	na	-0.01
*Cavy*6	8	0.600	0.790	5	0.333	0.641	6	0.542	0.676	6	0.583	0.822	8	0.615	0.794	0.31
*Cavy*7	3	0.000	0.514	3	0.111	0.582	3	0.333	0.591	3	0.067	0.545	4	0.118	0.364	0.86
*Cavy*8	4	0.286	0.261	2	0.000	0.192	3	0.240	0.389	3	0.200	0.191	2	0.167	0.157	0.29
*Cavy*9	3	0.235	0.670	2	0.500	0.500	3	0.240	0.493	3	0.071	0.505	3	0.111	0.565	0.68
*Cavy*10	4	0.368	0.596	2	0.125	0.125	4	0.150	0.550	3	0.250	0.540	2	0.125	0.484	0.60
*Cavy*11	4	0.267	0.736	2	0.000	0.667	4	0.125	0.442	4	0.167	0.712	3	0.143	0.385	0.72
*Cavy*12	5	0.158	0.589	4	0.182	0.249	4	0.000	0.550	3	0.000	0.304	2	0.000	0.129	0.86
*Cavy*14	4	0.150	0.558	5	0.444	0.760	3	0.059	0.508	3	0.167	0.591	3	0.083	0.475	0.74
*Cavy*15	3	0.286	0.577	2	0.000	0.667	4	0.138	0.564	4	0.313	0.683	5	0.263	0.690	0.65
*Cavy*16	4	0.571	0.632	na	na	na	3	0.480								
*Mean*	4.000	0.277	0.549	2.909	0.168	0.420	3.545	0.213	0.497	3.455	0.022	0.521	3.455	0.175	0.447	0.60
																
*SD*	1.549	0.182	0.197	1.221	0.179	0.26	1.036	0.177	0.151	1.036	0.208	0.223	1.809	0.16	0.224	0.221

N: sample size; Na: number of alleles; Ho: observed heterozygosity; He: expected heterozygosity; F: Inbreeding; na: not available. Wouri, Moungo and Nkongsamba are in the Littoral region, and Mémé and Fako are in the Southwest Region of Cameroon.

The observed and expected heterozygosity rates were calculated for each population under Hardy-Weinberg equilibrium. To estimate the importance of genetic polymorphism, observed and expected heterozygosity rates were compared. The expected rates were higher than those observed for all populations reflecting a heterozygote deficiency. The highest rate was 0.277 in the population of Wouri. These results were consistent with the rates of inbreeding observed for all loci studied, which were very high IN all studied populations (Table 1).

Table 2 shows the results of the HardyWeinberg equilibrium test for all loci studied and for all populations. The population of Nkongsamba presented a high balance compared to other populations. Outside the locus cavy 3, which was in equilibrium in all populations, all other deviated from that equilibrium. However, considering each subpopulation, there were also loci in balance with the Hardy-Weinberg equilibrium. This was the case of loci cavy 2, 3, 8, 11 for Fako, cavy loci 2, 3, 8 for Wouri; cavy 3, 6, 8, 10 and 14 for Mémé; and loci cavy 3 and 7 for Moungo, whereas in the population of Nkongsamba, only loci cavy 2, 7 and 8 showed a deviation from the Hardy-Weinberg equilibrium. The equilibrium observed for locus cavy 3 confirmed the absence of inbreeding at this locus, while the other loci showed a high rate of inbreeding and violations of equilibrium conditions.

**Table 2:** Test of significance of the Hardy-Weinberg equilibrium per locus and cavy population from Littoral and Southwest Regions of Cameroon (N = 109)

Locus	Littoral Region			Southwest Region	
	Wouri	Nkongsamba	Moungo	Mémé	Fako
*Cavy*2	ns	ns	***	***	***
*Cavy*3	ns	ns	ns	ns	ns
*Cavy*6	*	ns	**	ns	*
*Cavy*7	***	**	ns	**	**
*Cavy*8	ns	***	***	ns	ns
*Cavy*9	**	ns	***	***	***
*Cavy*10	*	ns	***	ns	**
*Cavy*11	**	ns	***	*	ns
*Cavy*12	***	ns	***	***	***
*Cavy*14	***	ns	***	ns	***
*Cavy*15	**	ns	***	***	***
*Cavy*16	***	ns	***	**	***

ns, not significant; *, **, and ***, significant at p ≤ 0.05, p ≤ 0.01 and p ≤ 0.001, respectively

Molecular variance assessed in the total study population is summarized in Table 3. A high variability was observed among individuals within each population (54.4%) and between individuals of the total population (40.6%), leaving only a small percentage (4.9%) of variation among populations.

**Table 3:** Analysis of molecular variance in the total cavy population studied from Littoral and Southwest Regions of Cameroon

Source of variation	Sum squares	Variance components	Percentage of variation
Among populations	33.41	0.14	4.92
Among individuals within populations	295.53	1.61	54.41
Within individuals	91,0	1.20	40.66
Total	419.95	2.96	

Among the five populations studied, those from Nkongsamba and Moungo had the greatest genetic distance but also the lowest genetic identity, respectively, 0.227 and 0.758 (Table 4). Populations from Mémé and Fako, both from Southwest Region, on the other hand, were those closest as having the greatest genetic identity and the lowest genetic distance (0.992 and 0.008, respectively). Low genetic distances observed among populations show that they were close and probably had a common history.

**Table 4:** Unbiased genetic distance and identity of Nei between different cavy populations from Littoral and Southwest Regions of Cameroon

	Populations				
	Wouri	Nkongsamba	Moungo	Mémé	Fako
Wouri		***0.226***	***0.101***	***0.031***	***0.049***
Nkongsamba	0.798		***0.277***	***0.174***	***0.220***
Moungo	0.904	0.758		***0.053***	***0.100***
Mémé	0.969	0.840	0.949		***0.008***
Fako	0.952	0.802	0.905	0.992	

Unbiased genetic distance are shown above the diagonal (indicated in bold) and identity of Nei is shown below the diagonal (N = 109)

### Structure and phylogenetic relationship between populations

**Figure 1:**
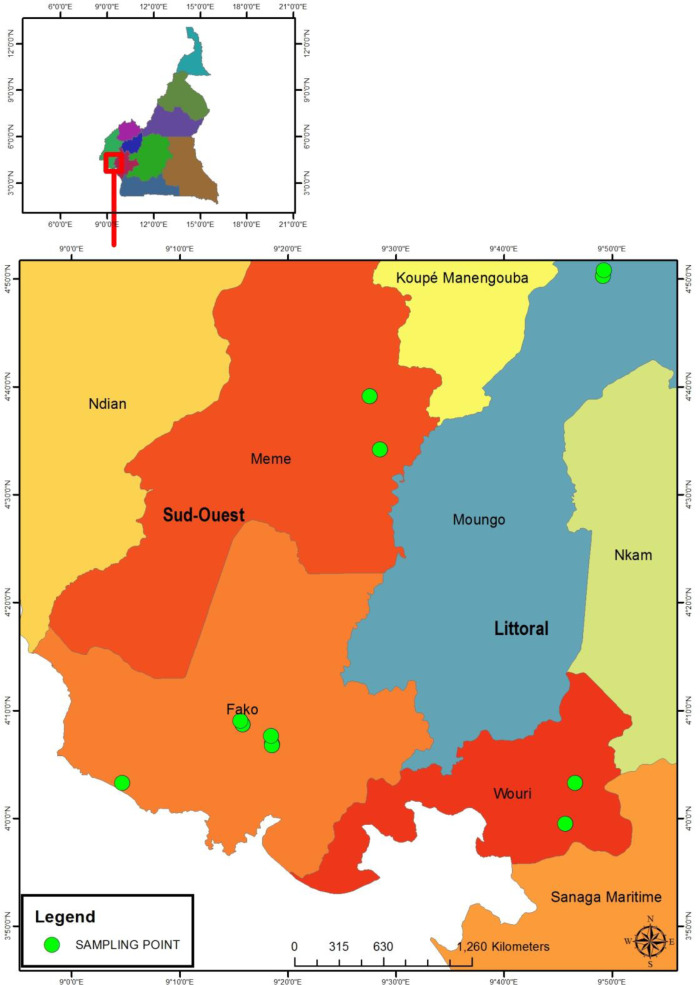
Household surveyed in the different regions of Southern Cameroon

The number of genetic populations was equal to K = 3 (Figure 1), the peak obtained following Evanno et al. (2005). Thus, the overall study population was considered to be composed of three distinct genetic types. Populations from Wouri and Moungo were composed of one dominant subpopulation (green) despite the presence of introgression, whereas other populations consisted of two subpopulations. Observed introgression implies that much of the distribution of individuals is done regardless of their geographical origin and, probably, uncontrolled crosses take place between existing genetic types.

**Figure 2:**
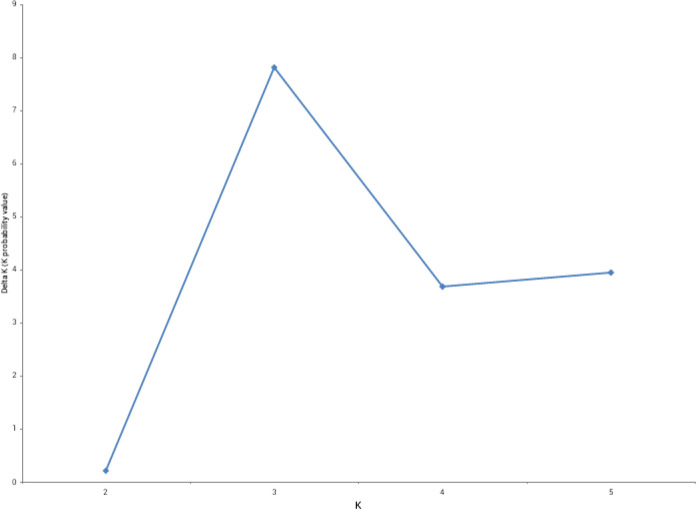
The uppermost hierarchical level of genetic partitioning among the overall cavy population from Littoral and Southwest Regions of Cameroon (N = 109) assessed according

**Figure 3:**
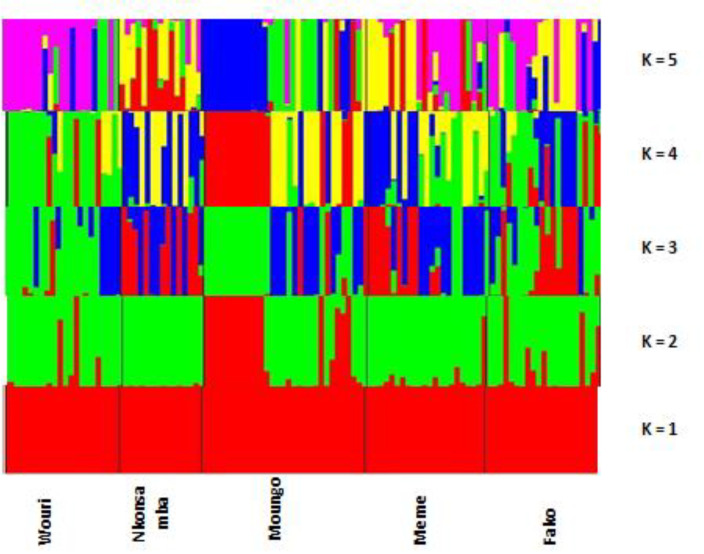
Genetic structure of the total cavy population from Littoral and Southwest Regions of Cameroon (N=109). The overall population is partitioned into K groups corresponding to the number of colors; black vertical lines separate the different populations studied.

### Discussion

This study reports, a study on genetic diversity and population structure of domestic cavies from Cameroon at molecular level. Seventeen alleles were observed in the total population with an average of 3.5 alleles per locus in five populations studied. The lowest number of alleles where observed in Nkongsamba ecotype (2.909) while Wouri had the greatest number (4.00). Poutougnigni et al. (2015) and Wikondji et al. (2015) obtained respectively 5.1 and 5.9 alleles per locus in population from forest and highland regions in Cameroun. Compared to Southwest region, number of alleles per ecotype from littoral region seems to be more variable. The overall number of alleles observed in this study was lower than that determined by Kouakou et al. (2015) who obtained on average 5.98 ± 0.37 alleles from Cote d’Ivoire cavy populations using the same markers; however, these authors sampled from large parts of the country, while this study focused on a much narrower geographic area in Cameroon. BurgosPaz et al. (2011) in three lines of domestic cavy from Colombia/South America, recorded on average 6.8 alleles per locus with a total of 34 alleles, when using 5 microsatellites; however, this number is close to that obtained in the tame line (4.8 alleles). Unsurprisingly, Kanitz et al. (2009) found an even higher average number of alleles of 8.5 and 6.3, respectively, for the wild species *Cavia magna* and *Cavia aperea*. The relatively low number of alleles observed in the current study could be due to the small sample size, assuming that an increase of the sampled population would result in an increase of the probability of finding new alleles (Foulley et al. 2006). In addition, the low number of alleles observed could also be related to mutations, deletions and a high level of inbreeding in the population. Finally, it is to be expected that only a fraction of genetic diversity available in South American domestic cavies has been introduced to Africa, whenever and how often this introduction has taken place.

In the whole studied populations, the expected heterozygosity was high than the observed heterozygosity. The same observation have been done by Wikondi et al. (2015) and Poutougnigni et al. (2015) in highland and forest cavy populations from Cameroon. Populations from Southwest region had the high deviation from expected heterozygosity (0.499 for Mémé and 0.302 for Fako) compared to those form littoral. Both the expected and observed heterozygosity values obtained by Kouakou et al. (2015), respectively, 0.619 ± 0.03 and 0.528 ±0.04 for expected and observed heterozygosity are higher than those reported in this study. The same conclusions are found considering the results from Kanitz et al. (2009) studying two wild cavies; *Cavia magna* and *Cavia aperea* (0.681 and 0.648, and 0.656 and 0.573, respectively). The high rate of inbreeding (0.60) observed in the studied populations could help explain the low rates of observed and expected heterozygosity. On the other hand, the largest deviation from expected heterozygosity (0.499 across all loci for Mémé) was lower than that reported by Burgos-Paz et al. (2011).

Except for locus cavy 3, all studied loci deviated from Hardy-Weinberg equilibrium in the general population. These results differ from those obtained by Kanitz et al. (2009), who observe a balance for all markers in *C. aperea*, but deviation in the loci cavy1, 5 and 10 in *C. magna*. Burgos-Paz et al. (2011) record a deviation from Hardy-Weinberg equilibrium at 4 out of 5 loci in three lines of domestic cavy in Colombia (native cavy, improved and tame cavy). These results show that, as long as there is restricted flock size, Hardy-Weinberg equilibrium is deviated in some loci.

The deviation from the equilibrium in this study can be explained by the fact that in Cameroon, the average size of a cavy population is 18 per household (Ngou Ngoupayou et al. 1995; Yiva et al. 2014), and this small flock size cannot lead to random mating. In addition, there is often exchange of animals among farmers within a region and even among regions.

The high rate of molecular variance observed between individuals from different populations and between individuals of the same population have been already reported as well by Wikondi et al. (2015) and Poutougnigni et al. (2015) in highland and forest cavy populations from Cameroon and would suggest there are opportunities for intra-population selection before crossing individuals from the different populations for possible genetic improvement of the species in Cameroon. Similar results have been found by Kouakou et al. (2015) who also recorded a small molecular variation among populations (2.6%), while within populations and individuals it was intermediate (22.0%) and large (75.4%) respectively. This could be related to low exchange of animals within Côte d’Ivoire compared to Cameroon.

The five populations in this study were structured into three distinct genetic groups. Evolutionary theory of populations can help explain these groups. According to Mohamed et al. (2010), the genetic constitution of a population has a possibility of variation over time, and there are forces that determine genetic variations of populations. The result of these evolutionary forces is to vary the rate of heterozygosity of the population compared to the Hardy-Weinberg equilibrium. In addition, two populations of the same species may be subject to different evolutionary factors, resulting in genetic divergence from one another over time, which can produce an effect on both allele frequencies and the relationship between observed and expected heterozygosity. Chauca (1997) noted that, in general, the population of cavy has preserved significant genetic and phenotypic variability.

A low level of genetic structure as observed in this study and a high rate of inbreeding has been reported from several production systems. This could be attributed to nonrandom mating and selection (e.g., Granevitze et al. 2007 for chicken; Kumar et al. 2006 for buffalo; Serrano et al. 2009 for goats; Wu et al. 2009 for ducks). As a short-cycle small livestock species, cavy is not different when considering its rapid growth, high reproductive rate and the traditional farming system in Cameroon. The species is susceptible to very high inbreeding rates as found in this study.

## Conclusion

The results of this study show that existing microsatellites developed for cavy populations in South America are useful tools for the genetic characterization of cavy populations in sub-Saharan Africa. They indicate that the studied populations from southern Cameroon have a satisfactory genetic diversity that can be used as a basis for conservation of and selection from this small livestock species to improve productivity. Future studies should expand to include cavy populations from northern and northwestern regions of Cameroon as well as those from other African countries. This may help not only to identify useful pockets of less related genetic diversity for improvement but also clarify the possible evolution of domestic cavy on the African continent.
